# Effects of Citric Acid on Structures and Properties of Thermoplastic Hydroxypropyl Amylomaize Starch Films

**DOI:** 10.3390/ma12091565

**Published:** 2019-05-13

**Authors:** Yang Qin, Wentao Wang, Hui Zhang, Yangyong Dai, Hanxue Hou, Haizhou Dong

**Affiliations:** 1 Department of Food Science and Engineering, Shandong Agricultural University, 61 Daizong Street, Tai’an 271000, China; qyghostz@sdau.edu.cn (Y.Q.); wwtlxm@126.com (W.W.); zhanghui@sdau.edu.cn (H.Z.); dyyww@sdau.edu.cn (Y.D.); 2 Engineering and Technology Center for Grain Processing of Shandong Province, Tai’an 271000, China

**Keywords:** hydroxypropyl amylomaize starch, citric acid, cross-linking, reactive extrusion, biodegradable plastic film

## Abstract

Hydroxypropyl amylomaize starch (HPAS) films were prepared by hot press. The effects of initial pH of HPAS on the mechanical properties, molecular interaction, structure, and cross-linking degree of the resultant films were investigated. A weak acidic condition was suitable for cross-linking of citric acid and HPAS by reactive extrusion. The film of HPAS with an initial pH of 5.66 had the maximum tensile strength of 7.20 MPa and elongation-at-break of 94.53%, and the weight average molecular weight of HPAS increased to 4.17 × 10^5^ g/mol. An appropriate initial pH facilitated the formation of diester bonds between HPAS and citric acid during extrusion, but too low initial pH levels resulted in hydrolysis of starch molecules and reduced the mechanical properties.

## 1. Introduction

Starches are promising materials for the preparation of biodegradable plastic and their industrial application can reduce the environmental costs of petroleum-derived materials [1]. Thermoplastic starch films show better gas barrier performance than polyethylene films, therefore, they are suitable for using as inner packaging of foods, such as nuts, fruits, and vegetables [2]. 

However, the poor processability of starch is one of the main reasons that limited the application of starch film. Natural starch granule has a hierarchical periodic structure formed by alternating amorphous and semicrystalline growth rings [3]. This unique lamellar structure makes starch a nonthermoplastic material as the pyrolysis point is lower than the melting point of the crystalline regions [4]. Even some plasticizers (e.g., urea, water, glycerol, glycol, sorbitol, and sugars) are added into starch to disrupt the semicrystalline structure [5]; the starch films remain brittle and have poor mechanical properties compared to low-density polyethylene [6]. 

Some studies have shown that the films prepared from amylomaize starch had higher tensile strength than those from normal maize starch [3,7]. Nevertheless, the high gelatinization temperature and melt viscosity of amylomaize starch have limited its practical application [8]. Thus, reducing gelatinization temperature and melt viscosity by chemical modification is an effective method to improve its film-forming ability. 

By disrupting the hydrogen bonds in the starch granules, hydroxypropyl groups render amylomaize starch more likely to gelatinize at lower temperature [9] with better clarity and less syneresis [10]. Hydroxypropyl amylomaize starch (HPAS) is suitable for preparing the films with better flexibility and mechanical properties by solution casting [6]. Generally, hydroxypropyl starch is prepared by reacting a kind of starch, including amylomaize starch, with propylene oxide at the presence of sodium hydroxide [11]. Moreover, cross-linking after hydroxypropylation of high amylose starch can significantly improve high-temperature resistance of starch [12]. 

Citric acid (CA) is a trifunctional carboxylic acid and has been used in the production of cross-linked starch [1]. The addition of citric acid can improve the polysaccharide’s thermal tolerance and water stability while inhibiting the film’s retrogradation during storage by forming strong hydrogen and ester bonds with starch molecules [13]. It has also been reported that citric acid had a positive effect on the plasticization and melt processing properties of starch [14]. However, the introduction of citric acid in some cases can substantially reduce the tensile strength of the starch films while improving elongation-at-break [13]. Citric acid also hydrolyzes starch as a side reaction that is influenced by pH, reaction temperature, and citric acid concentration while cross-linking takes place [15].

In this study, hydroxypropyl high amylomaize starch with non-neutralized sodium hydroxide has been used as film-forming initial material. Different amounts of citric acid were added as acid to adjust the pH of hydroxypropyl high amylomaize starch and cross-linking agent to prepare cross-linking starch by reactive extrusion. The effects of different citric acid amounts on the properties of hydroxypropyl high amylomaize starch film have been investigated. The mechanism of performance improvement of hydroxypropyl high amylomaize starch films with citric acid had been discussed. 

## 2. Materials and Methods 

### 2.1. Materials

Amylomaize starch with 70% amylose was purchased from Ingredion China Limited (Shanghai, China). Dimethyl sulfoxide (DMSO), citric acid, and glycerol (AR) were purchased from Tianjin Kaitong Chemical Reagent Co., Ltd. (Tianjin, China). Deionized water was prepared using an AK Exceed-D lab pure water system (Chengdu Tangshi Kangning Technology Co., Ltd., Chengdu, China). 

### 2.2. Preparation of Hydroxypropyl High Amylomaize Starch (HPAS)

High amylomaize starch was mixed with 2% NaOH powder (based on dry starch mass) for 5 min at room temperature. Then, 25% anhydrous ethanol and 10% epoxypropane (based on dry starch mass) were slowly added into the mixture in a reactor. The reactor was heated up to 80 °C by steam and mechanically stirred for 4 h. The hydroxypropyl high amylomaize starch with degree of substitution of 0.3 was obtained after cooling to room temperature. The hydroxypropyl high amylomaize starch was not neutralized after hydroxypropylation and the pH was 10.92 for 1% suspension. 

### 2.3. Preparation of HPAS-CA Films

A certain amount of citric acid was dissolved in glycerol. Non-neutralized hydroxypropyl amylomaize starch was thoroughly mixed with citric acid–glycerol solution in a SHR-50A high-speed mixer (Hongji Co., Ltd., Zhangjiagang, China). The 1 g mixture of hydroxypropyl amylomaize starch, glycerol and citric acid was dispersed in 100 mL deionized water and stirred for 30 min at room temperature. The pH of the suspension was determined by FE-20 pH Meter (Mettler Toledo LLC., Shanghai, China). The sample codes and relevant sample information were presented in Table 1. In this work, the HPAS-10.92 film was the control film that no citric acid was added. 

Thermoplastic HPAS-CA composite was obtained by extrusion using a SHJ-20B co-rotating twin-screw extruder (Nanjing Giant Machinery Co., Ltd., Nanjing, China). The extruder barrel temperature profile was 85-90-100-105-100-95 °C from the feeder to the die end. The screw rotation speed was 150 rpm. The thermoplastic HPAS-CA composite was cut into pellets after cooling to room temperature.

A hot press technique was used to prepare the HPAS-CA films. About 16 g of the thermoplastic HPAS-CA pellets was placed between two 20 cm × 20 cm stainless steel plates completely covered with a tetrafluoroethylene sheet at 120 °C for 2 min. The pressure was then raised and held at 10 MPa for an additional 7 min. To reduce the thickness of the films, a pressure of 30 MPa was applied for 5 min, after which the mold was cooled to 35 °C using cold water circulation. The HPAS-CA films were stored in an HWS-60 constant temperature and humidity chamber (Shanghai Jinghong Laboratory Equipment Co., Ltd., Shanghai, China) at 23 °C and 53% relative humidity for 72 h.

### 2.4. Degree of Diesterification of HPAS-CA Films 

The degree of diesterification (DDE) between citric acid and HPAS was determined based on the complexometric titration of citric acid with copper (II)-sulfate [16]. Two independent titrations were carried out on the HPAS-CA films: (1) The HPAS-CA films were dissolved in deionized water and titrated to measure the free and monoesterified citric acid. (2) Potassium hydroxide (KOH) was used to hydrolyze the cross-links in the HPAS-CA films to measure the total amount of citric acid.

The HPAS-CA film of 300 mg was cut into pieces, and totally dissolved either in 50 mL of deionized water or in 50 mL of 0.1 M KOH for 20 min in a boiling water bath. The pH of the solutions was then adjusted to 8.5 with acetic acid and borax/boric acid buffers at room temperature. The solution was made up to 250 mL and titration was carried out with 0.02 M copper sulfate where in 1 mL of copper (II) solution consumed 3.842 mg of citric acid. Murexide (Aladdin Industrial Corporation, Shanghai, China) was used as the indicator. The difference between the titration of the two film solutions enabled the calculation of DDE and diester proportion of total citric acid [16]. All measurements were performed in triplicate.

### 2.5. Thickness

The thicknesses of the HPAS-CA nanocomposite films were determined using 211-101F micrometers (Guilin Guanglu Measuring Instrument Co., Ltd., Guilin, China). All the films were tested randomly, and the thicknesses of the HPAS-CA films were calculated by averaging the results of six replicate measurements.

### 2.6. Mechanical Properties

The tensile strength (TS, MPa) and elongation-at-break (EAB, %) of the HPAS-CA films were determined with an XLW (PC) Auto Tensile Tester (Labthink Instruments Co., Ltd., Jinan, China) in accordance with ASTM D882-12 [17]. Each treated film was cut into strips (50 mm × 15 mm) prior to test. The initial distance between the top and bottom clamps was 100 mm and the test speed were 100 mm/min. The TS and EBA of each film were obtained by averaging the results of the six replicates.

### 2.7. Attenuated Total Reflectance Fourier-Transform Infrared Analysis

Fourier-transform infrared (FT-IR) analysis was performed on the HPAS-CA films using a Nicolet iS 5 spectrometers with an attenuated total reflectance (ATR) accessory (Thermo Fisher Scientific, Waltham, MA, USA) over a wavenumber range of 4000 to 400 cm^−1^. The number of accumulated scans and the scanning rate were 32 and 4 cm^−1^, respectively. To better identify the vibrations of hydroxypropyl high amylomaize starch and citric acid that significantly overlap with one another, differential spectra and deconvolution were performed at the bands that are relative to the hydroxypropyl high amylomaize starch short-range order structure and esterification between hydroxypropyl high amylomaize starch and citric acid using Peakfit software version 4.0 (Systat Software Inc, San Jose, CA, USA).

### 2.8. ^13^C Solid-State Nuclear Magnetic Resonance

The ^13^C solid-state nuclear magnetic resonance (^13^C SSNMR) was performed using a Bruker Avance III 400 MHz wide bore spectrometer (Bruker-AXS, Karlsruhe, Germany) equipped with a 4-mm broadband double-resonance cross-polarized magic angle spinning probe. Each film (500 mg) was placed onto the rotor and introduced to the center of a magnetic field. The spectra were decomposed using the Peakfit software.

### 2.9. Molecular Weight of HPAS-CA Films 

One milligram of HPAS-CA film was dissolved into 1 mL dimethyl sulfoxide (DMSO) with 50 mM LiCl and stirred for 2 h at 90 °C. The solution was then magnetic-stirred for 8 h at room temperature and filtered through a 1.0-μm nylon-66 Millipore filter (Jinteng experimental equipment Co. Ltd., Tianjin, China).

Static light scattering (SLS) was employed to measure the weight average molecular weight (M_w_) of HPAS-CA film using a BI-200SM research goniometer and laser light scattering system (Brookhaven Instruments Corporation, Holtsville, NY, USA). The light source was a laser (λ = 633 nm). The dn/dc value was 0.147. The tests of SLS were performed in the angle range of 30 to 140° in steps of 5°. The M_w_ of the various HPAS-CA films were calculated using the Berry method.

### 2.10. Scanning Electron Microscopy

The surfaces of HPAS-CA films were analyzed with the scanning electron microscope (SEM) FEI QUANTA FEG25 (FEI company, Hillsboro, OR, USA). The surfaces were vacuum coated with gold for SEM and the magnification was 2000 times.

### 2.11. X-Ray Diffraction

The crystallinity and crystal structure of the HAS film were tested by X-ray diffraction (XRD) using a D8 Advance X-ray diffractometer (Bruker-AXS, Karlsruhe, Germany). The Cu target line was used, λ = 0.15406 nm, 2θ = 5–40°, and the step frequency was 0.02°/s.

### 2.12. Statistical Analysis 

The data in tables and figures are represented in terms of mean values ± standard deviation (SD). Any significant differences in the group mean values, at the 95% significance level, were tested using analysis of variance (ANOVA), followed by post-hoc Duncan’s multiple range tests using SPSS software 21 (IBM Co., Armonk, NY, USA).

## 3. Results and Discussion

### 3.1. Appearance of HPAS-CA Films

**Figure 1:**
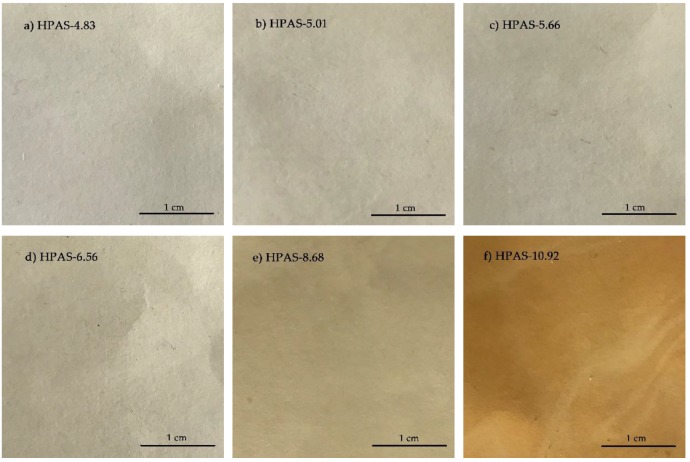
Images of HPAS-CA films with different citric acid amount.

With the decrease of initial pH of hydroxypropyl high amylomaize starch, the appearance of starch film changed from brown to colorless (Figure 1). The presence of un-neutralized NaOH caused the oxidative decomposition [18,19] of hydroxypropyl high amylomaize starch during extrusion and browned the HPAS-10.92 film. With adding citric acid, the NaOH in hydroxypropyl high amylomaize starch was neutralized and alkali hydrolysis was inhibited, the HPAS-CA film became colorless with increasing citric acid amount.

### 3.2. Mechanical Properties of HPAS-CA Films 

**Figure 2:**
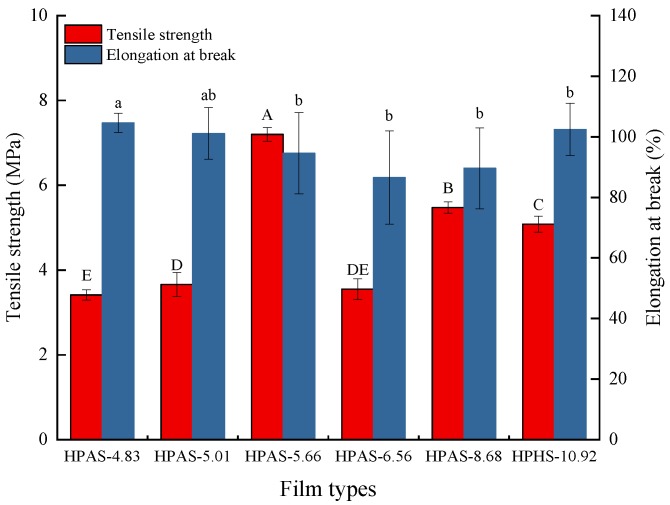
Tensile strength and elongation-at-break of HPAS-CA films with different citric acid amount. Bars sharing the same letter are not significantly different (*p* < 0.05, same in the other figures).

The elongation at break (EAB) values of the HPAS-CA films slightly changed with different initial pH of starch (Figure 2). However, the citric acid had an obviously effects on the TS values of HPAS-CA films. The HPAS-10.92 film had a TS of 5.08 MPa (Figure 2). With the addition of citric acid, the TS increased to 5.47 MPa at a pH of 8.68. When the initial pH was 6.56, i.e., near neutrality, the TS decreased by almost 35% (3.55 MPa) with the addition of only 0.25% more citric acid into the hydroxypropyl high amylomaize starch The TS value of the film had a significant increase when the pH decreased to 5.66. The TS of the HPAS-5.66 film was 7.20 MPa, and this was the maximum among all the samples. The increase of the tensile strength (TS) value might be attributed to the cross-linking reaction between citric acid and hydroxypropyl high amylomaize starch, which reinforced the film [20]. The further decrease of initial pH, the acidolysis dominated the film-forming process, and resulted in a sharp fall in TS. The TS was 3.66 MPa for the HPAS-5.01 film and 3.41 MPa for the HPAS-4.83 film. 

### 3.3. Thickness of HPAS-CA Films 

**Figure 3:**
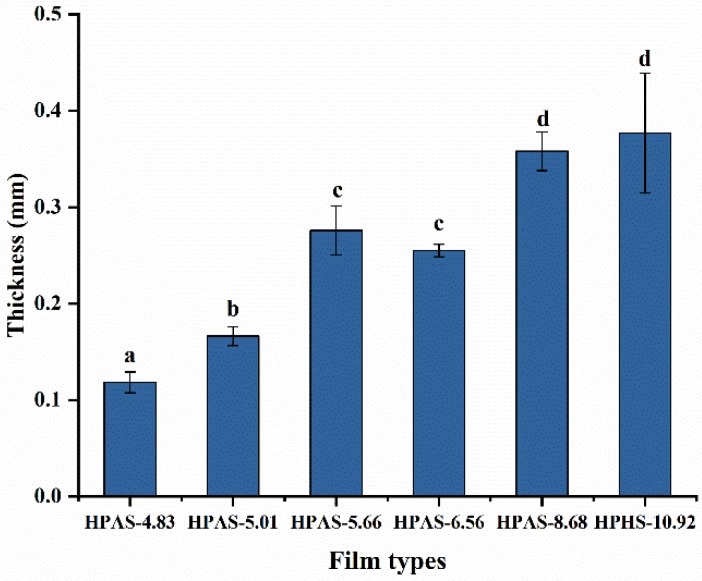
Thickness of HPAS-CA films with different citric acid amount.

The thickness of HPAS-CA films significantly decreased with the decrease of the initial pH of starch. As the mass of the pellets and the processing parameters of hot press were identical across all samples, the addition of citric acid appeared to soften the thermoplastic hydroxypropyl high amylomaize starch, rendering it easier to be pressed into films (Figure 3). The HPAS-5.66 film had an abnormal thickness, which might be attributed to high cross-linking degree with low mobility. 

### 3.4. Morphology of HPAS-CA Films

**Figure 4:**
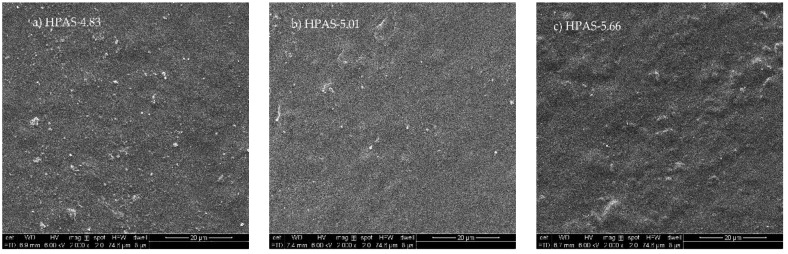

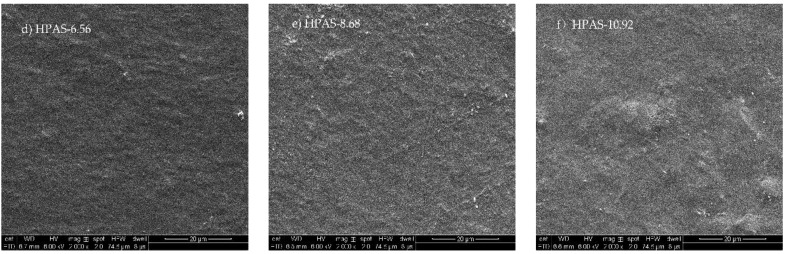
SEM images of HPAS-CA films with different citric acid amount at 2000× magnification.

The morphology of HPAS-CA films is shown in Figure 4. The HPAS-10.92 showed a rough surface with large area of agglomerates and several residual granular structures. By adding citric acid, the surfaces of HPAS-8.68 and HPAS-6.56 films were smoother than that of HPAS-10.92. This indicated that citric acid promoted the fragmentation and plastify of hydroxypropyl high amylomaize starch granules. However, the morphology of HPAS-CA films was rough again in weak acid condition. The HPAS-5.66 film had a surface with small area of agglomerates that might be attributed to the cross-linking reaction between hydroxypropyl high amylomaize starch and citric acid. The granular structures on the surface of HPAS-4.83 film were the recrystallized particles formed by acidolysis [13,21].

### 3.5. Crystalline Structure of HPAS-CA Films 

**Figure 5:**
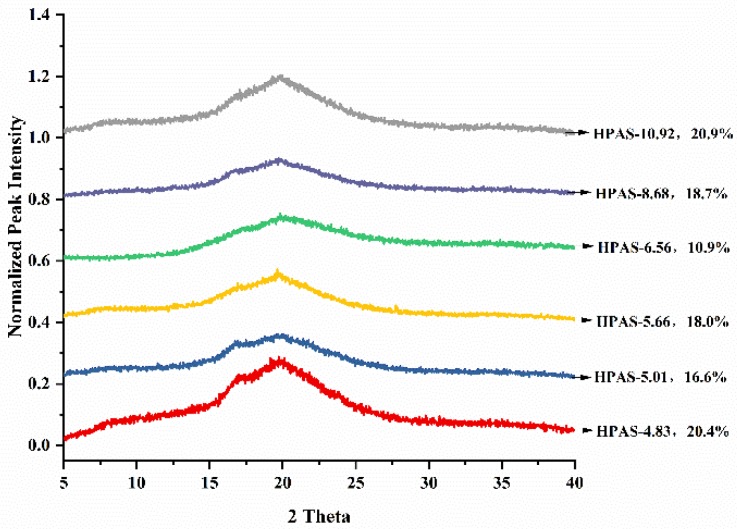
XRD patterns of HPAS-CA films with different citric acid amount.

The XRD patterns of HPAS-CA films with different pH values are shown in Figure 5. All samples had a broad peak at 2θ = 14–26° indicated that the crystal structure of hydroxypropyl high amylomaize starch was almost destroyed in the film-forming process. The crystallinity of HPAS-CA films decreased from 20.9% (HPAS-10.92) to 10.9% (HPAS-6.56), which confirmed that citric acid prompted the gelatinization of hydroxypropyl high amylomaize starch and inhibited its recrystallization [22]. However, as the pH further decreased, the crystallinity was increased to 18.0%, 16.6%, and 20.4% for HPAS-5.66, HPAS-5.01, and HPAS-4.83, respectively. The reason for the irregular change of crystallinity could be attributed to the combined action of cross-linking reaction and acidolysis in HPAS-CA film with various citric acid content. In HPAS-5.66 film, the diester structure formed by the cross-linking reaction between hydroxypropyl high amylomaize starch and citric acid, inhibited the gelatinization of HPAS and the movement of molecular chains and resulted in the increase of crystallinity [23,24]. With lower pH condition, acidolysis of citric acid dominated the film-forming process and caused the degradation of starch crystal structure, decreasing the crystallinity [25]; conversely, the accumulated short chain fragments formed by acidolysis recrystallized and formed quasi-crystalline structure, which increased the crystallinity of HPAS-CA film. This could be a reasonable explanation for the raised crystallinity of HPAS-4.83 and the appearance of the weak peaks at 2θ = 19° and 20° [26].

### 3.6. Molecular Interaction between Hydroxypropyl High Amylomaize Starch and Citric Acid

**Figure 6:**
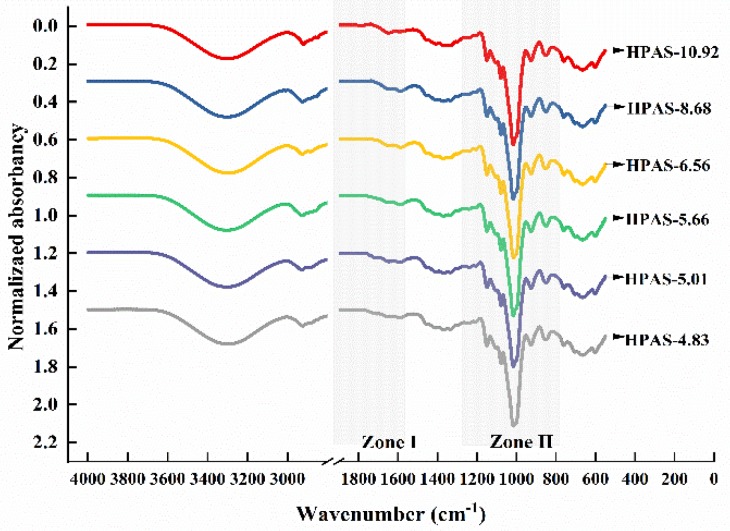
Attenuated total reflectance Fourier-transform infrared (ATR-FTIR) spectra of HPAS-CA films with different citric acid amount.

ATR-FTIR spectroscopy was employed to investigate the interactions between hydroxypropyl high amylomaize starch and citric acid in HPAS-CA films. Although all the films had similar spectra, differences appeared mainly in two zones (Figure 6). Zone I was the weak bands at ~1500–1800 cm^−1^, correlating with sodium citrate, ester group, including monoester and diester, formed by hydroxypropyl high amylomaize starch and citric acid, and carboxyl group [27,28].

**Figure 7:**
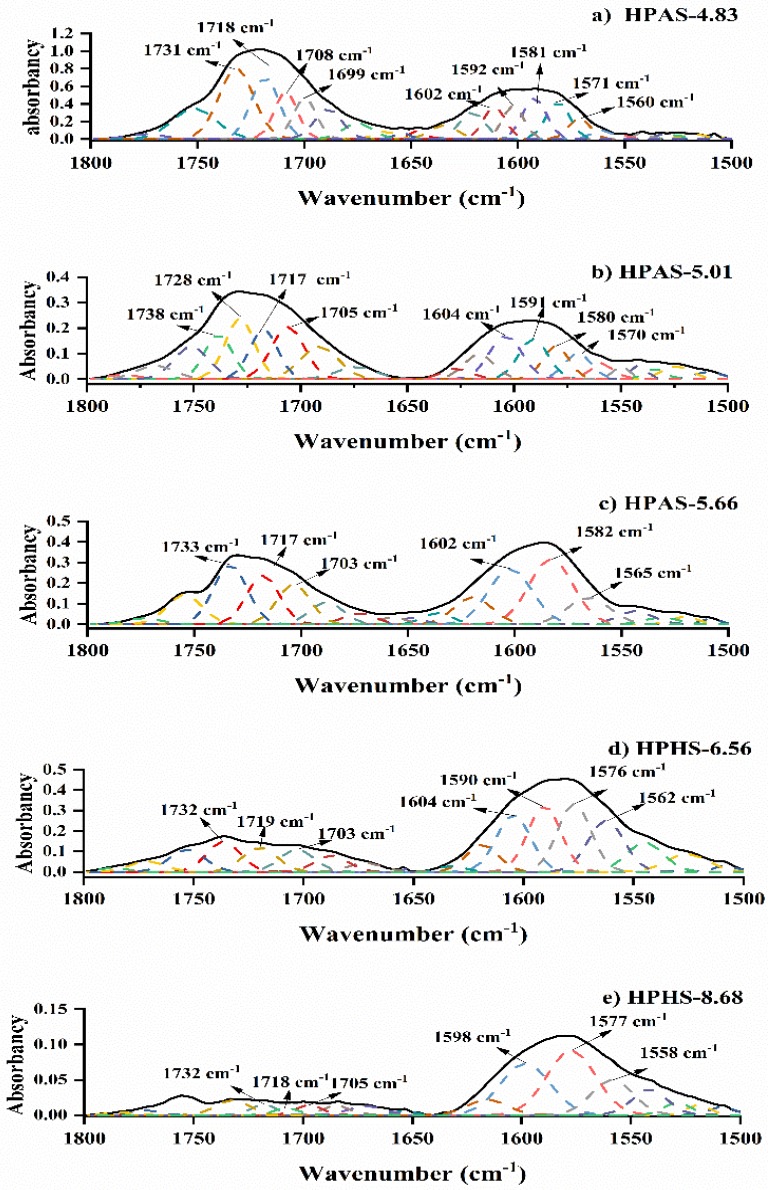
Fitting of peaks to the HPAS-CA films at ~1500–1800 cm^−1^: The experimental spectra are denoted by (—) and fitted peaks by (– – –).

After subtracting each spectrum of the HPAS-CA film from the HPAS-10.92 film, the resultant spectra showed obvious changes (Figure 7). The C=O stretching vibration of the carboxyl group was located at 1709 cm^−1^. The peak at 1732 cm^−1^ could be attributed to the esterification between citric acid and hydroxypropyl high amylomaize starch during reactive extrusion [1]. The band at ~1500–1600 cm^−1^ correlated with a carboxylate–metal ion interaction, including CA^−^, CA^2−^, and CA^3−^ [27]. When pH decreased from 8.68 to 4.83, the intensity of the peak at 1560 cm^−1^ had a significantly decreased that could be suspected as the citric acid molecular changed from fully deprotonated to doubly protonated. The strong peak at 1577 cm^−1^ in the spectrum of the HPAS-8.68 film (Figure 7e) indicated that the citric acid molecule was mainly present as CA^3−^, which could be used as acid to neutralize sodium hydroxide. Meanwhile, there were a few citric acid molecules in the CA^2−^ state, which is indicated by the peaks at 1598 cm^−1^ and 1558 cm^−1^, that could esterified with starch and brought about a weak peak at 1732 cm^−1^ [29]. In the HPAS-6.56 film spectra, the equally correlated peaks of CA^2−^ and CA^3−^ suggested that the amounts of the two ions were almost the same. Also, greater esterification between starch and citric acid occurred, as the intensity of the peak at 1732 cm^−1^ was stronger than that in HPAS-8.68. A further decrease in pH made the citric acid more prevalent in the states of CA^2−^ and CA^−^, and this helped increase the formation of ester bonds between HPAS and citric acid, as the bands at around 1700 cm^−1^ became stronger in the HPAS-5.66, HPAS-5.01 and HPAS-4.83 films spectrum when compared with those of HPAS-6.56 and HPAS-8.68. This indicated that lower pH promoted esterification between citric acid and starch during reactive extrusion. 

The bands at 800–1200 cm^−1^ (Zone II) were correlated with the stretching peaks of C–C and C–O in the starch molecule, which was sensitive to changes in the short-range structure during extrusion and hot pressing [30]. The hydroxy in the C–OH group of the intact starch molecule could form a hydrogen bond with the oxygen of the C–O–C group, and its characteristic peak was located at 1022 cm^−1^. After extrusion and esterification between citric acid and hydroxypropyl high amylomaize starch, the weakening of the interaction between the starch molecules resulted in the C–OH peak shifting to 1018 cm^−1^ [31]. This peak was considered to come from an amorphous part in the starch granule. Correspondingly, the peak at 1039 cm^−1^, which should be located at 1047 cm^−1^ in an intact starch molecule, represented the organized part of the starch in the HPAS-CA film. The ordered and amorphous starch structure at short range was measured by the ratio of the intensity of the peak at 1039 cm^−1^ to that at 1018 cm^−1^ (R_1039/1018_) (Table 2). As the local order under the formation of helical fragments might be stabilized by inter- and intra-HPAS molecule hydrogen bonds [32]. The increase of the value of R_1039/1018_ suggested that the addition of citric acid into hydroxypropyl high amylomaize starch promoted the formation of inter-hydrogen bonds and reduced the free volume available for starch mobility, resulting in more rigid films [6]. However, acidolysis might degrade the HPAS molecule during extrusion, which led the value of R_1039/1018_ to decrease when the initial pH of hydroxypropyl high amylomaize starch decreased to 5.01 and 4.83. 

### 3.7. Complexometric Titration of Citric Acid with Copper (II)-Sulfate 

In all the HPAS-CA films, the proportion of diester ranged from 2.57% to 21.45% of the total citric acid (Table 2). The HPAS-5.66 film had the highest diester concentration of 21.45%, and this proportion decreased as pH decreased from 5.66 to 4.83. However, the film with the maximum proportion of diesters did not have the highest DDE, as the DDE value continued to increase as pH decreased. The increase in DDE value implied that the increasing amount of citric acid in the HPAS-CA film was beneficial to the formation of the diester cross-linking structure [15]. However, the proportion of diester to total citric acid in HPAS-CA films was relatively low which indicated that citric acid tends to form more monoester with HPAS and acidolysis occurred in lower pH condition.

### 3.8. Weight Average Molecular Weight of HPAS-CA Films 

The combined action of cross-linking and acidolysis resulted in various M_w_ of HPAS-CA films (Table 2). The M_w_ of HPAS-10.92 film was 3.79 × 10^5^ g·mol^−1^. The increased M_w_ of HPAS-5.66 film indicated that citric acid formed intermolecular cross-linking bonds with hydroxypropyl high amylomaize starch during thermal extrusion [16]. The HPAS-5.66 film had a maximum M_w_ of 4.17 × 10^5^ g·mol^−1^. The M_w_ of HPAS-CA films decreased as the initial pH of hydroxypropyl high amylomaize starch decreased which could be attributed to the acidolysis of citric acid. 

**Figure 8:**
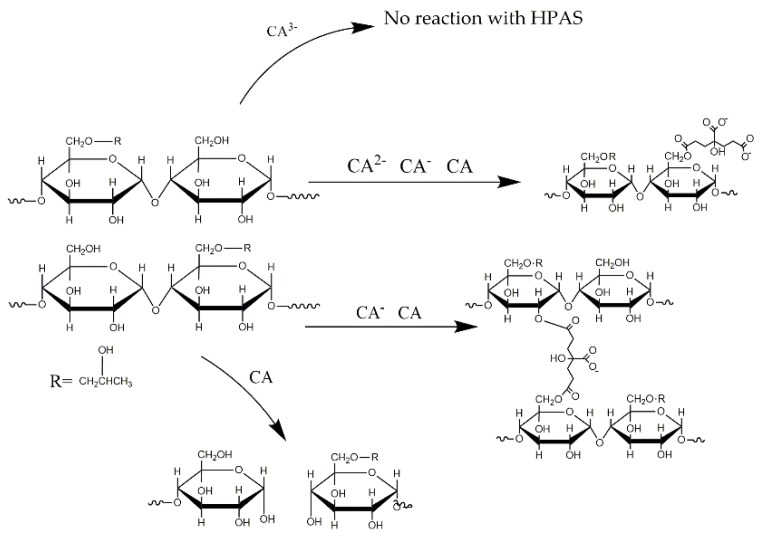
Reaction of citric acid and hydroxypropyl high amylomaize starch under various pH by melting extrusion.

The hypothesis of the reaction between citric acid and hydroxypropyl high amylomaize starch under various pH by melting extrusion was shown as Figure 8. Under the alkaline condition, the citric acid would prior to react with excessive NaOH and dissociates into CA^3−^. This prevented the reaction between citric acid and starch molecule. With the increasing of amount, which lead a decrease of the pH, citric acid might dissociate into CA^2−^ and CA^−^. Both of them could form an ester bond with starch; the CA^−^ and the undissociated citric acid could form diester bond with starch that was also called as cross-linked reaction. However, under the acidic condition, the excessive citric acid provided enough carboxyl to react with the hydroxy [15]. Then the water produced by esterification might lead acidolysis which decreased the molecular weight of hydroxypropyl high amylomaize starch.

### 3.9. Short-Ordered Structure of HPAS-CA Films 

**Figure 9:**
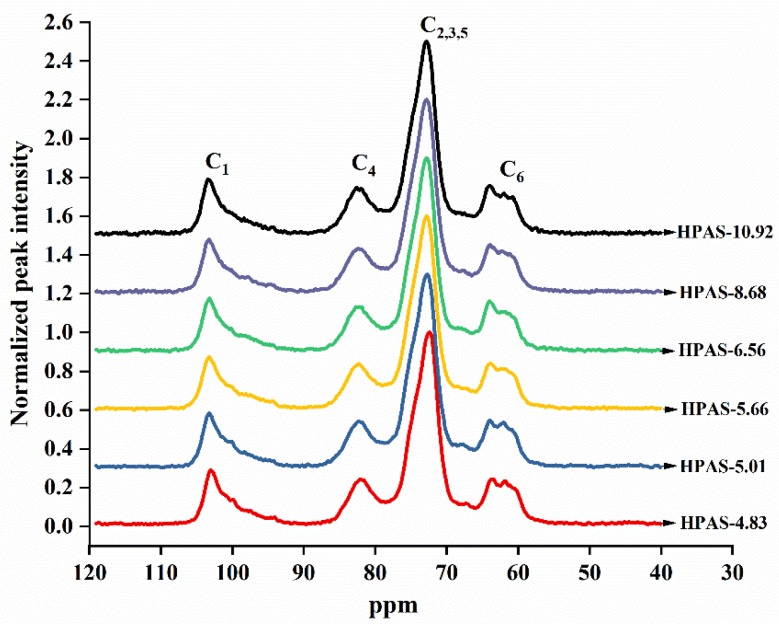
^13^C solid-state nuclear magnetic resonance (SSNMR) spectrum of HPAS-CA films with different citric acid amount.

The ^13^C-SSNMR spectra of the HPAS-CA films were similar to each other (Figure 9). The region between 80 ppm and 65 ppm was formed by a series of peaks, which was due to poor resolution of the solid state. This region was commonly correlated with C_2_, C_3_, and C_5_ carbons, and C_2_, C_3_, and C_5_ with substitution groups such as the hydroxypropyl and ester groups in modified starch [33]. Although the C_1_ and C_4_ carbon did not carry hydroxyl groups or cross-link with citric acid, they were influenced by the groups substituted on C_2_, C_3_, or C_6_, and parts of them shifted to the low field [33]. The proportions of single and double helices were calculated using the subspectrum of the C_1_ region (90–105 ppm). The ordered spectrum at the C_1_ peak could be resolved into the single helix V-type component (102–103 ppm) and the double helices component (99–102 ppm). The spectrum at the C_4_ peak was related to the amorphous structure of the HPAS-CA films. The relative proportions of single helix, double helices, and amorphous structures in the six kinds of the HPAS-CA films are shown in Table 3. 

Compared to HPAS-10.92 film, the HPAS-8.68 film had greater amorphous area and double helices but lower single helix area. This indicates that the addition of citric acid neutralized the alkali which might play a role in transforming the double helices structures into single helix ones [34]. The citric acid promoted the gelatinization of HPAS during extrusion and the cross-linking reaction, which maintained the single helix conformation of amylose [35]. This was the reason for the increase of single helix area from HPAS-8.68 to HPAS-5.66 film. In the case of the HPAS-5.66, HPAS-5.01, and HPAS-4.83 films, the amorphous area decreased as more citric acid was added to HPAS, indicating that rigid structures of ester and diester were formed between the starch chains [36]. However, the single helix area in HPAS-CA films decreased as initial pH of HPAS decreased from 5.66 to 4.83, indicating that the acidolysis of citric acid also promoted the degradation of the single helix structure during extrusion. The recrystallization of the small, short chain fragments formed by acidolysis of starch increased the double helix area [26]. These findings agreed with the XRD results. 

## 4. Conclusions

The cross-linking reaction between citric acid and hydroxypropyl high amylomaize starch was achieved by reactive extrusion while preparing thermoplastic starch. The HPAS-CA film with initial pH at 5.66 had tensile strength at 7.20 MPa and elongation at break at 94.53%. The tensile strength of the film had an obvious decrease as the pH decreased. Citric acid would prefer neutralize NaOH at alkaline condition and promoted the gelatinization of hydroxypropyl high amylomaize starch during reactive extrusion. The weak acid condition would be more suitable for esterification and produce diester cross-linked structure between hydroxypropyl high amylomaize starch and citric acid and reinforced the film. The acidolysis was enhanced as pH further decreased which degraded starch molecule and reduced the properties of starch film.

## Figures and Tables

**Table 1:** Sample codes and relevant sample information.

Sample Codes	HPAS/g	Glycerol/g	Citric Acid/g	pH of 1% HPAS-CA Suspension
HPAS-4.83	2000	600	120	4.83
HPAS-5.01	2000	600	100	5.01
HPAS-5.66	2000	600	80	5.66
HPAS-6.56	2000	600	45	6.56
HPAS-8.68	2000	600	40	8.68
HPAS-10.92	2000	600	0	10.92

**Table 2:** Diesterification (DDE), R_1039/1018_, and M_w_ of the HPAS-CA films.

Sample	R_1039/1018_	Proportion of Diester to Total CA/%	DDE of Starch/×10^−3^	M_w_ /×10^5^ g·mol^−1^
HPAS-4.83	0.544	17.78 ± 3.14	7.0 ± 1.22	3.18 ± 0.15
HPAS-5.01	0.544	18.63 ± 3.20	6.0 ± 1.04	3.44 ± 0.09
HPAS-5.66	0.637	21.45 ± 5.99	5.6 ± 1.55	4.17 ± 0.23
HPAS-6.56	0.610	15.60 ± 1.04	2.3 ± 0.15	4.12 ± 0.16
HPAS-8.68	0.562	2.57 ± 0.20	0.3 ± 0.03	3.81 ± 0.14
HPAS-10.92	0.548	0	0	3.79 ± 0.18

* The values in the table are expressed as average ± SD.

**Table 3:** Proportions of single helix, double helices, and amorphous structures of HPAS-CA films with different citric acid amount.

Sample	C_1_		C_4_
	Single Helix Area/%	Double Helix Area/%	Amorphous Area/%
HPAS-4.83	24.33	24.79	50.88
HPAS-5.01	33.08	19.23	47.69
HPAS-5.66	34.43	15.43	50.14
HPAS-6.56	27.04	20.26	52.70
HPAS-8.68	23.89	23.54	52.57
HPAS-10.92	27.69	21.10	51.21

## References

[1] Zhou J., Tong J., Su X., Ren L. (2016). Hydrophobic starch nanocrystals preparations through crosslinking modification using citric acid. Int. J. Biol. Macromol..

[2] Wang W. (2016). Preparation, Film-Forming Mechanism and Application of Starch-Based Nanocomposite Films. Ph.D. Thesis.

[3] Li M., Liu P., Zou W., Yu L., Xie F., Pu H., Liu H., Chen L. (2011). Extrusion processing and characterization of edible starch films with different amylose contents. J. Food. Eng..

[4] Nafchi A.M., Moradpour M., Saeidi M., Alias A.K. (2013). Thermoplastic starches: Properties, challenges, and prospects. Starch-Stärke.

[5] Zdrahala R.J. (1997). Thermoplastic starch revisited. Structure/property relationship for “dialed-in” biodegradability. Macromol. Symp..

[6] Kim H.-Y., Jane J.-L., Lamsal B. (2017). Hydroxypropylation improves film properties of high amylose corn starch. Ind. Crop. Prod..

[7] Muscat D., Adhikari B., Adhikari R., Chaudhary D. (2012). Comparative study of film forming behaviour of low and high amylose starches using glycerol and xylitol as plasticizers. J. Food. Eng..

[8] Thuwall M., Boldizar A., Rigdahl M. (2006). Extrusion processing of high amylose potato starch materials. Carbohydr. Polym..

[9] Wootton M., Manatsathit A. (1983). The Influence of Molar Substitution on the Water Binding Capacity of Hydroxypropyl Maize Starches. Starch-Stärke.

[10] Gunaratne A., Corke H. (2007). Effect of hydroxypropylation and alkaline treatment in hydroxypropylation on some structural and physicochemical properties of heat-moisture treated wheat, potato and waxy maize starches. Carbohydr. Polym..

[11] Lee H., Yoo B. (2011). Effect of hydroxypropylation on physical and rheological properties of sweet potato starch. LWT-Food Sci. Technol..

[12] Tessler M.M., Jarowenko W., Amitrano R.A. (1975). Hydroxypropylated, Inhibited High Amylose Retort Starches. U.S. Patent.

[13] Yu J., Wang N., Ma X. (2005). The effects of citric acid on the properties of thermoplastic starch plasticized by glycerol. Starch-Stärke.

[14] Wang N., Yu J., Han C. (2007). Influence of citric acid on the properties of glycerol-plasticised cornstarch extrusion blends. Polym. Polym. Compos..

[15] Olsson E., Menzel C., Johansson C., Andersson R., Koch K., Järnström L. (2013). The effect of pH on hydrolysis, cross-linking and barrier properties of starch barriers containing citric acid. Carbohydr. Polym..

[16] Menzel C., Olsson E., Plivelic T.S., Andersson R., Johansson C., Kuktaite R., Järnström L., Koch K. (2013). Molecular structure of citric acid cross-linked starch films. Carbohydr. Polym..

[17] ASTM (2012). D882-12 Standard Test Method for Tensile Properties of Thin Plastic Sheeting.

[18] Golova O.P., Nosova N.I. (1973). Degradation of Cellulose by Alkaline Oxidation. Russ. Chem. Rev..

[19] Tang M., Wen S., Liu D. (2016). Effects of heating- or caustic-digested starch on its flocculation on hematite. Miner. Process. Extr. Metall. Rev..

[20] Reddy N., Yang Y. (2010). Citric acid cross-linking of starch films. Food Chem..

[21] Ortega-Toro R., Collazo-Bigliardi S., Talens P., Chiralt A. (2016). Influence of citric acid on the properties and stability of starch-polycaprolactone based films. J. Appl. Polym. Sci..

[22] Morales N.J., Candal R., Famá L., Goyanes S., Rubiolo G.H. (2015). Improving the physical properties of starch using a new kind of water dispersible nano-hybrid reinforcement. Carbohydr. Polym..

[23] Mei J.-Q., Zhou D.-N., Jin Z.-Y., Xu X.-M., Chen H.-Q. (2015). Effects of citric acid esterification on digestibility, structural and physicochemical properties of cassava starch. Food Chem..

[24] Li M.-N., Xie Y., Chen H.-Q., Zhang B. (2019). Effects of heat-moisture treatment after citric acid esterification on structural properties and digestibility of wheat starch, A- and B-type starch granules. Food Chem..

[25] Chen P., Xie F., Zhao L., Qiao Q., Liu X. (2017). Effect of acid hydrolysis on the multi-scale structure change of starch with different amylose content. Food Hydrocoll..

[26] Sun X., Yu J., Liu Y. (2004). Study of acid hydrolysis course and properties of different starches. Fine Chem..

[27] Ramos M.E., Huertas F.J. (2014). Adsorption of lactate and citrate on montmorillonite in aqueous solutions. Appl. Clay Sci..

[28] Shi R., Bi J., Zhang Z., Zhu A., Chen D., Zhou X., Zhang L., Tian W. (2008). The effect of citric acid on the structural properties and cytotoxicity of the polyvinyl alcohol/starch films when molding at high temperature. Carbohydr. Polym..

[29] Strathmann T.J., Myneni S.C. (2004). Speciation of aqueous Ni(II)-carboxylate and Ni(II)-fulvic acid solutions: Combined ATR-FTIR and XAFS analysis. Geochim. Cosmochim. Acta.

[30] Van Soest J.J., Tournois H., De Wit D., Vliegenthart J.F. (1995). Short-range structure in (partially) crystalline potato starch determined with attenuated total reflectance Fourier-transform IR spectroscopy. Carbohydr. Res..

[31] Spiridon I., Teaca C.-A., Bodirlau R. (2011). Preparation and characterization of adipic acid-modified starch microparticles/plasticized starch composite films reinforced by lignin. J. Mater. Sci..

[32] Véchambre C., Buléon A., Chaunier L., Jamme F., Lourdin D. (2010). Macromolecular Orientation in Glassy Starch Materials That Exhibit Shape Memory Behavior. Macromolecules.

[33] Lawal O.S. (2009). Starch hydroxyalkylation: Physicochemical properties and enzymatic digestibility of native and hydroxypropylated finger millet (*Eleusine coracana*) starch. Food Hydrocoll..

[34] Wang S., Copeland L. (2012). Effect of alkali treatment on structure and function of pea starch granules. Food Chem..

[35] El-Tahlawy K., Venditti R.A., Pawlak J.J. (2007). Aspects of the preparation of starch microcellular foam particles crosslinked with glutaraldehyde using a solvent exchange technique. Carbohydr. Polym..

[36] Tan I., Flanagan B.M., Halley P.J., Whittaker A.K., Gidley M.J. (2007). A Method for Estimating the Nature and Relative Proportions of Amorphous, Single, and Double-Helical Components in Starch Granules by13C CP/MAS NMR. Biomacromolecules.

